# Insights from the 2023 International Veterinary Immunology Symposium: global perspectives at Kruger National Park

**DOI:** 10.1186/s13567-024-01409-4

**Published:** 2024-11-26

**Authors:** Alejandra V. Capozzo, Yolanda Corripio-Miyar, Yolandy Lemmer

**Affiliations:** 1 CONICET-Universidad Abierta Interamericana. Centro de Altos Estudios en Ciencias Humanas y de la Salud, Montes de Oca 745, Buenos Aires, Argentina; 2 Moredun Research Institute, Pentlands Science Park, Bush Loan, Penicuik, Midlothian EH260PZ UK; 3 Future Production: Chemicals, CSIR, Pretoria, 0002 South Africa

**Keywords:** veterinary immunology, networking, research achievements, research gaps, vaccination

## Abstract

The 13th International Veterinary Immunology Symposium (IVIS) was initially due to take place in August 2022, but as many things in our lives, the COVID-19 pandemic that hit the world two years prior, forced the organising committee to postpone the meeting until November 2023. As it is tradition, the veterinary immunology symposium was organised as a satellite meeting of the IUIS International Congress of Immunology, which in 2023 took place in Cape Town, and it is where veterinary immunologists from all over the world get together to discuss advances and challenges in the field of animal health. The 2023 International Veterinary Immunology Symposium (IVIS) was held from November 17th to 21st at Kruger National Park, Skukuza, South Africa. This was the first time the symposium was hosted on the African continent. This event gathered 210 veterinary professionals and scientists from 38 countries to discuss the latest advancements and challenges in veterinary immunology. A highlight of the event was that over 70% of the delegates were first-time attendees, contributing to the symposium’s global reach. The symposium featured a series of 83 oral presentations and 104 poster presentations, including topics relating to protective immunity, vaccine strategies, important disease targets, and methodological advancements in veterinary immunology. Workshops provided hands-on experiences and discussions on new technologies such as next-generation sequencing and vaccine development strategies against bacterial infections. The symposium also provided opportunities for networking and engagements with leaders in the field, set against the backdrop of one of Africa’s most iconic game reserves, enhancing the experience with a unique blend of professional exchange and natural beauty.


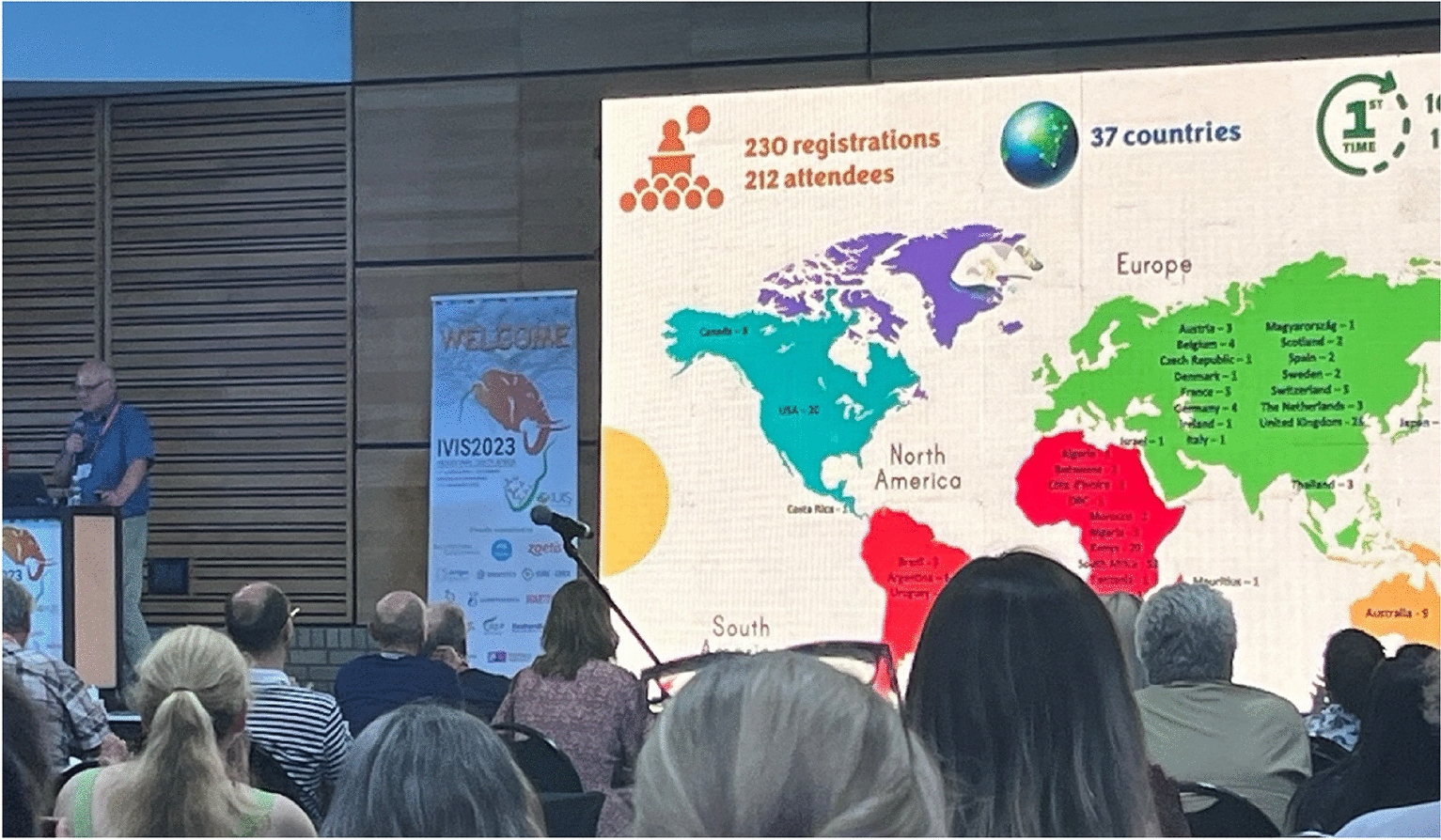


*The IVIS 2023 in Kruger was attended by 212 delegates from 37 countries*.

Over 5 days, with 5 workshops and up to three parallel sessions, many different areas of veterinary immunology were covered. Here is an overview of what was discussed in the meeting and the workshops.

## Workshops

The five different workshops presented attracted a lot of interest and discussions from the delegates at the symposium. The first two workshops were held before the official opening of the symposium and included the Next-generation sequencing and transcriptomics workshop led by Prof. Christine Maritz-Olivier of the University of Pretoria. The challenges of working in Africa were highlighted throughout her talk—as it was in many other talks throughout this meeting. Christine also pointed out the difficulty of finding bioinformaticians in Africa. Details around the advantages of single-cell sequencing were explained aiming at the early career scientists and how these techniques could reveal the heterogeneity within a cell population, enabling the identification of distinct cell types, states, and lineages. Sample collection in remote areas where Foot and Mouth Disease (FMD) is present, requires high biosecurity measures and consequently, sample collection as well as processing must be carried out in the field.

Examples of how single-cell and spatial multi-omics are empowering exciting new research into autoimmunity, immuno-oncology, and vaccine development were also presented. Representatives of distributors and application specialists in this field presented some of the platforms used in this field such as Illumina, MGI, PacBio, Oxford Nanopore Technologies (ONT, nanopore sequencing) as well as the best applications for these technologies, and how many researchers are now moving away from bulk RNA sequencing and onto single cell sequencing. Single cell sequencing was showcased in a series of talks which highlighted the many applications of this technology, such as the identification of novel cell subtypes and potential roles in the nutrient absorption in dairy cattle cross-tissue single-cell transcriptomics [1]. Single cell sequencing has also been used in bovine milk or porcine peripheral blood mononuclear cells (PBMC) [2]. Dr Crystal Loving, from the USDA, having used the 10 × platform with porcine cells, emphasized the importance of the platform selection and experimental design. Questions such as which single cell sequencing represent our cell of interest, which tissue will be most appropriate or how will the data be validated, are key. At the same time, when experiments are set up appropriately, single cell sequencing can lead to great results like the identification of novel cell types [3]. Long read sequencing (PacBio and ONT) can also be used in combination with single cell sequencing to better identify different isoforms as highlighted in one of the talks where human cells were sequenced and demultiplex into 12 M unique transcripts using the 10X Genomics platform from around 3000 PBMC [4]. These talks were complemented with a tutorial on how to navigate the Loupe Browser v7.0, an online resource to visualise 10X Genomics Chromium and Visium data.

The second workshop was dedicated to the “*Novel approaches to the development of veterinary vaccines to control bacterial infections*” and was chaired by Prof. Gary Entrican and Prof. Jayne Hope from the Roslin Institute in Scotland. Prof. Paul Wood, from Monash University (Australia), opened a series of short talks highlighting the importance of the use of Target Product Profile (TPP), a strategic planning document used to guide vaccine development process from the earliest stages. Outer membrane vesicles (OMVs) were discussed in the context of bacterial vaccines. OMVs are naturally produced by bacteria and can be used as a flexible adjuvant platform as well as vaccine carriers. A good example of this technology is the vaccine against meningococcal serogroup B, 4CMenB. This vaccine, formulated for human use, contains four highly immunogenic antigens which combined with OMVs, provides cross-protection towards multiple MenB strains. Prof. Brendan Wren from the London School of Hygiene & Tropical Medicine (LSHTM) introduced the use of bacterial glycoconjugate vaccines in livestock and the recently created spin-out company ArkVax. Bioconjugation consists of a protein component covalently linked to a glycan antigen. This methodology for vaccine production is less complex than traditional chemical conjugation, as well as cheaper and more flexible to produce as they showcased with a Mycoplasma vaccine they are currently working on. The use of viral vectors for vaccine production, a platform which is more advanced in veterinary species than it is in humans, was then discussed by Prof. Michael Jarvis, from the Vaccine Group in the UK. Viral vectors have many advantages, they are highly safe, they are cheap to produce, and requires no adjuvants in the formulation as they are inherently immunostimulatory molecules. They can be very useful when requiring protection from multiple serotypes as is the case of *Streptococcus suis*, a disease which currently relies heavily on the use of antibiotics for its control. The last platform discussed was mRNA vaccines by Helba Bredell from Afrigen Biologics, in Cape Town. There are two types of mRNA vaccines: self-amplifying and non-amplifying. The latter is a conventional mRNA vaccine which uses positive stranded RNA viruses to express the antigen of interest. However, these vaccines are unstable and easily degraded by the host cell machinery. Self-amplifying vaccines on the other hand, are less costly and have been shown to enhance antigen expression at lower doses. They use a positive stranded virus where structural proteins are substituted by the gene of interest. Once in the host cell, the viral vaccine can amplify the antigen encoding mRNA. This mimics a viral infection, resulting in sustained levels of antigen production combined with the stimulation of innate immune responses (adjuvant effect) which can lead to long lasting immunity. The Rift valley fever virus is a good example of a self-amplifying mRNA vaccine.

Prof. Gary Entrican introduced STAR-IDAZ, an international consortium which aims to coordinate research on animal health, with deliverables including candidate vaccines, diagnostics, therapeutics and other animal health products, along with procedures and key scientific information/tools to support analysis and disease control. Dr Johannes Charlier, from Kreavet in Belgium, concluded the short presentations discussing Discontools, a program focused on identifying gaps in knowledge of aspects of infectious diseases such as diagnostics, vaccines and pharmaceuticals.

The third of the workshops was a regular at the IVIS meetings, the *Immunological Toolkit session*, led by Prof. Jayne Hope from the Roslin Institute where a few speakers provided an overview of ongoing work in reagent development and novel recombinant antibodies driving immunological research. The workshop started with a series of short talks on novel antibodies such as anti-porcine CD38, the use and production of recombinant antibodies or novel methods for sequencing and annotating cattle antibodies. Two talks highlighted the work carried out by the Immunological Toolbox at the Roslin Institute (RI) and Pirbright Institute (PI). Dr Inga Dry, from RI, gave an example of the antibodies they are producing against ADGRE1, the large animal homologue of mouse F4/80 and human EMR1. While Prof. John Hammond (PI) focused on the work they have been doing sequencing B-cell receptors of historical hybridoma cell lines and determining if the resulting monoclonal antibodies have retained their antigen-specificity. This not only secures the future availability of these antibodies, but it also offers the opportunity of engineering recombinant antibodies. Following the talks, facilitated discussions took place where the attendants had the opportunity to share their requirements for reagent availability/use as well as any difficulties they might experience in using the reagents. One of the challenges highlighted included the dissemination of information, for instance, many people were not aware of the existence of the Immunological Toolbox and the services they offer to the research community. A recurrent issue we also face when working with veterinary species is not just the limited availability of antibodies, but also the poor selection of commercially available fluorochromes and conjugated antibodies. The access of reagents in developing countries is another great challenge which was highlighted not only during these discussions, but also throughout this symposium. Storage of cell lines, difficulties sharing reagents and lack of funding were also amongst the issues discussed.

In a similar format of short talks and discussion, the *VIC-MHC workshop* took place on the third day of the conference following the immunological toolkit session. Here a comparative analysis of swine leukocyte antigen (SLA) in Gӧttingen minipigs and commercial pig lines was presented alongside an overview of the latest update of the SLA complex annotation. Two more talks discussed the non-classical MHC-I haplotypes in ruminants and the role of non-polymorphic MHC-I and innate-like T cells in *Xenopus* tadpoles during resistance and tolerance to Mycobacteria. The session finished with an update on the IPD-MHC Database, a centralised repository for MHC sequences from different taxonomic groups of veterinary importance, from fish to ruminants or dogs.

The final workshop of the symposium related to supporting of early career researchers in veterinary immunology and vaccinology research as was hosted by Dr Tim Connelley representing the International Veterinary Vaccinology Network (IVVN). Presentations from representatives of Afrique One Aspire, Bill and Melinda Gates Foundation (BMGF), The African Academy of Sciences (AAS) and The African Research Universities Alliance (ARUA) were given, focusing on ways in which early career researchers could be assisted in developing and growing their careers. Throughout the session, several talks offered information on a variety of programs focused on the development of young scientists in the African continent. The African Research Initiative for Scientific Excellence Pilot Programme (ARISE) is an example of these initiatives. They support fellowships for early to mid-career researchers from African countries for a period of 5 years. Rowland Opisa, from the African Academy of Science (AAS), highlighted how African institutions struggle with governance and how, even though the AAS have been working hard to ameliorate these issues, it could inevitably have an impact in some of these fellowships. Another African focused initiative is the Afrique One-African Science Partnership for Intervention Research Excellence (ASPIRE), which focuses on research on endemic zoonotic diseases supporting fellowships from the masters level all the way to post-doctoral level. The workshop concluded with brief talks from some of the IVVN fellows, a fellowship program aimed at researchers from LMICs, where they shared their experiences during their fellowships.

## Highlights from the scientific program

The conference highlights are listed in Table 1, with the challenges that were identified during the discussions.

**Table 1:** **Themes highlighted in the meeting and identified gaps**

Highlighted themes	Advances	Gaps and challenges
Avian Influenza Vaccines	• Virus-like particle vaccines demonstrated high immunogenicity and efficacy [7–10] • Regular updates to vaccines for antigenic matching to field viruses	• Control of Highly Pathogenic Avian Influenza (HPAI) • Current reliance on stamping out H5 and H7 outbreaks
Bat immunology	• Understanding bat antiviral immunity and unique immunological traits [13] • Mechanisms of bats as viral reservoirs and their potential to prevent outbreaks	Understanding bat-host dynamics • Detailed molecular pathogenesis and disease reservoir mechanisms • Impact of dietary changes on antibody repertoire and viral immunity
Porcine Disease Vaccines	• Innovative vaccine strategies for *Streptococcus suis* [11, 12] • Research on immunological responses to PRRS, ASF, and swine influenza	Efficacy of porcine vaccines • Absence of efficacy studies • Lack on basic research into germinal center disruption and IgG function
Next-Generation Sequencing	• Advances in single-cell sequencing for identifying cell heterogeneity • Applications in autoimmunity, immuno-oncology, and vaccine development	• Specific training needed for young scientists based on LMIC and improve access to equipment
Veterinary Vaccine Platforms	• Novel vaccine technologies for bacterial infections • Focus on deployment in low- and middle-income countries (LMICs)	• Affording accessible technologies for veterinary use
Research development in Africa	• Limited veterinary professionals and infrastructure hindering growth • Lack of targeted investments to expand the veterinary industry	• Need for structured support in career development and growth • Enhanced funding and mentorship opportunities for young scientists

Veterinary immunology plays a vital role in the overall health management of animal populations, safeguards human health, and contributes to our understanding of immunological principles across species. One of the primary objectives of the meeting was to highlight challenges and advancements made from an African perspective in the veterinary industry. One of the keynote speakers, Prof. Baptiste Dungu from the University of Kinshasa and Design Biologix, highlighted some challenges and innovative approaches for vaccine development and production in Africa.

As indicated by market reports, the veterinary industry in Africa, while growing, is relatively small compared to other regions globally. The global veterinary medicine market is substantial, with market sizes reported in the tens of billions of U.S. dollars. In contrast, while specific statistics for Africa’s share of the global market are not typically detailed in general industry reports, it is known that the region faces unique challenges such as a shortage of veterinary professionals and infrastructure, which can hinder growth and therapeutic development. Additionally, the veterinary markets in North America and Europe are more mature and significantly larger, driven by higher pet ownership rates, greater spending on animal healthcare, and advanced veterinary services. Overall, the veterinary industry in Africa is expanding but remains a small fraction of the global market, influenced by regional economic conditions, livestock management needs, and the emerging market for companion animal care [5, 6].

Basic advances in veterinary immunology were also presented during the meeting. A study using transcriptomic analysis (presented by Prof. Bert Devriendt from Belgium) revealed the presence of a novel liver Natural Killer cell subset in swine, providing new insights into resident NK cells in this species. Tissue immunology studies were also addressed using precision-cut tissue slices, as presented by the group of Prof. Dirk Werling at the Royal Veterinary College, UK. These tissue culture studies, performed without serum, could be applied to study the specific inflammatory response triggered by *Mycoplasma hyopneumoniae* in pig lungs.

Regarding applied immunity, a study by Prof. Alistair Noble from The Pirbright Institute, UK, explored distinct effector functions mediated by Fc regions of bovine IgG subclasses and their interaction with Fc gamma receptors. This study revealed differences in bovine IgG subclass functionality compared to those of humans, mice, and pigs, which should inform future vaccine and therapeutic antibody development.

The theme of vaccine development was highly represented in both the oral and poster sessions, and several presenters showcased new and novel vaccines developed in the field.

Immunological studies on vaccine efficacy are crucial both after infection and vaccination, particularly for understanding protective immunity to achieve herd immunity. This was emphasized in discussions on viruses with high economic impact, such as African Swine Fever (ASFV) and Foot-and-Mouth Disease Virus (FMDV). Dr Ediane Silva from PIADC presented on the cellular and humoral immune response of pigs vaccinated with a live attenuated CSFV strain. Dr Alejandra Capozzo from Buenos Aires highlighted post-vaccination monitoring for FMDV, stressing the need for species-specific assays. She noted that ELISA tests validated for cattle may not be suitable for buffaloes and that a high-throughput ELISA measuring anti-FMD antibody avidity at a single dilution can be as accurate as traditional virus neutralization tests. This underscores the need for tailored field assays for effective monitoring.

## Zoonotic diseases and disease control: the One Health Perspective

Veterinary immunology is also essential for understanding how diseases affect animals and developing methods to prevent and treat these diseases. This includes the development of vaccines which are critical in preventing infectious diseases that can affect both domestic and wild animal populations. In her keynote address, Prof. Celia Abolnik from the University of Pretoria discussed the detrimental effects of Avian influenza on the continent. South Africa provides a good illustration of the challenges many countries worldwide face with avian influenza control. A low pathogenicity H6N2 has circulated endemically in the national poultry flock for decades, despite using a traditional chemically inactivated whole virus vaccine grown in eggs. On the other hand, wild aquatic birds introduced low pathogenicity H5N2 or H7N6 precursor viruses that mutated to high pathogenicity avian influenza (HPAI) in ostriches or chickens, respectively, and more recently, clade 2.3.4.4b H5Nx HPAI viruses as part of an ongoing pandemic. Many countries including South Africa do not allow vaccination against HPAI and rather try to control H5 and H7 outbreaks through stamping out, but the clade 2.3.4.4b H5Nx HPAI pandemic is becoming more widespread, severely impacting biodiversity and livestock production, and is potentially zoonotic. Vaccination may soon be the only option to stabilise global food security, protect endangered wild populations and prevent more deaths. Avian influenza vaccines must be updated regularly to ensure that they are antigenically matched to circulating field viruses, to prevent clinical signs, and mortality, and to prevent spread. As an example, she showcased virus-like particle vaccines as being highly immunogenic, efficacious and safe, and offering many other benefits over the traditional method of producing chemically inactivated vaccines grown in eggs [7–11]. A change in attitudes towards vaccination against avian influenza is required. This topic was also continued during the poster presentations from different laboratories where innovative vaccines against Avian influenza were proposed.

The recent advances in vaccine research were featured throughout the meeting, from the use of reverse vaccinology to design vaccines against Babesia, to systems immunology to identify immune responses to viral infections in pigs. Dr Teresa Freire, from the Universidad de la República in Uruguay, draw our attention to the importance of knowing the infection status prior to vaccinating animals. She showed how when animals were vaccinated against a respiratory virus or FMD during an infection with the liver parasite *Fasciola hepatica*, humoral immune responses to the vaccines are significantly reduced. However, when the animals were treated with anthelminthics, the immune response to the vaccine was restored. This highlights the importance of a holistic health management and, how unawareness of the animal’s health status prior to treatment can have major impacts on immunisation.

The theme of the immunology of diseases and potential vaccines in porcine was a key topic at the meeting and was highlighted by the keynote speaker, Prof. Mariela Segura from the Université de Montréal. She discussed interesting findings on how the zoonotic bacterium *Streptococcus suis* affects immunological responses and pointed out the challenges of managing disease outbreaks due to the absence of effective vaccines. Her team’s research indicated that *S. suis* disrupts the immune system by impacting germinal centres and potentially optimal IgG function. Consequently, IgM and potentially anti-capsular polysaccharide antibodies might serve as effective vaccine strategies to combat *S. suis* infections in the future [12, 13]. One of the plenary speakers at the symposium, Dr Wilhelm Gerner from the Pirbright Institute, focused on local adaptive immunity in pigs on how the balance between effector and regulatory T cells can influence the inflammation and damage caused by bacterial and viral pathogens during infections and experimental immunizations. Additional research in the field that was presented included diseases such as Porcine Reproductive and Respiratory Syndrome (PRRS), African Swine Fever (ASF), and swine influenza. Efforts included the understanding of the immunology of the diseases as well as the development of vaccines and management practices to prevent and mitigate outbreaks.

The rise of highly zoonotic viral infections such as Hendra virus, Nipah virus, Marburg virus, Ebola virus, Rabies virus, Middle East respiratory syndrome coronavirus, SARS-CoV, and the recent SARS-CoV-2 has driven significant advancements in bat research. These viral outbreaks have all been linked to various bat species. An interesting discussion around this topic was presented by the plenary speaker, Dr Michele Baker from CSIRO in Australia, where she highlighted some research into the antiviral immunity in bats. Bats have unique immunological traits that enable them to act as potential viral reservoirs. They are also capable of defending themselves against viruses and sustaining their immunity. Consequently, a comprehensive understanding of bat-virus biology is crucial to uncover the key factors that facilitate the coexistence and transmission of these viruses that could mitigate potential outbreaks in the future [14]. For example, Prof. Anca Dorhoi’s group from the University of Greifswald has done extensive research into bat molecular pathogenesis and the mechanisms on how these animals serve as disease reservoirs. Additional research presented on bat research included the interesting discussion of Dr Dan Crowley from the Plowright lab at Cornell University, where they showed that the low-affinity antibody repertoire towards viruses could be manipulated with experimental dietary changes [15].

## Symposium conclusion and future directions

The symposium concluded on a positive note with a keynote address by Dr Samuel Thevasagayam, Deputy Director of Global Development of the Bill & Melinda Gates Foundation. Dr Thevasagayam highlighted the foundation’s commitment to an inclusive agricultural transformation aimed at increasing rural incomes, empowering women, improving family nutrition, and driving economic growth across the African continent. This commitment includes financial support for projects that enhance animal health, production, and nutrition. Another key area of interest is investing in the aquaculture industry, which provides up to 40% of animal-source food intake in Asia and 30% in Africa [16].

The symposium ended with a consensus on the critical need for enhanced international cooperation in addressing zoonotic diseases and advancing vaccine development. A recommendation was made for increased funding in veterinary research to support these initiatives. The importance of continuing education for veterinary professionals was also emphasised to ensure they remain current with rapid technological advancements.

Awards were given to posters selected by the Scientific Committee and the audience (Table 2).

**Table 2:** **Poster awards: the symposium recognized outstanding contributions through poster awards**

Theme	Awardee	Country	Title
Adaptive Immunity	Milena Brunner	Germany	Unravelling signals important for early chicken B-cell development
Immunology of Viral Diseases	Teerawut Nedumpun	Thailand	Different macrophage polarization patterns induced by porcine reproductive and respiratory syndrome viruses (PRRSV)
Vaccine Development	Anna Lacasta	Kenya	New advances in the development of a subunit vaccine targeting antibody production against African swine fever
	Ellen Cottingham,	Australia	Virally vectored immuno-contraceptives for the management of feral cats in Australia
Immunology of Wildlife and Exotics (Attendee’s Choice Award)	Camila Espejo,	USA	Dexamethasone increases in vitro immune cell proliferation in response to *M. bovis* in African buffalo

The organisers announced that the next IVIS meeting will be held in Austria in 2025. This upcoming event will focus on the latest advancements and innovations in veterinary immunology, continuing the tradition of fostering global collaboration and progress in the field.

## General conclusion

The 2023 IVIS highlighted significant advancements in veterinary immunology while identifying critical research gaps, particularly in infrastructure and vaccine development. The symposium emphasised the necessity for international collaboration and increased funding to address these challenges effectively. The next IVIS meeting in Austria 2025 aims to continue this progress, focusing on the latest innovations in the field.
